# Carcinogen Metabolism Pathway and Tumor Suppressor Gene Polymorphisms and Gallbladder Cancer Risk in North Indians: A Hospital-Based Case-Control Study

**DOI:** 10.31557/APJCP.2019.20.12.3643

**Published:** 2019

**Authors:** Takao Asai, Yasuo Tsuchiya, Kumudesh Mishra, Anu Behari, Pooja Shukla, Toshikazu Ikoma, Vinay K Kapoor, Kazutoshi Nakamura

**Affiliations:** 1 *Department of Clinical Engineering and Medical Technology, Niigata University of Health and Welfare, *; 2 *Division of Preventive Medicine, Niigata University Graduate School of Medical and Dental Sciences, Niigata, *; 4 *Division of Clinical Laboratory, Uji Tokushukai Medical Center, Uji, Kyoto, Japan, *; 3 *Department of Surgical Gastroenterology, Sanjay Gandhi Post Graduate Institute of Medical Sciences, Lucknow 226014 Uttar Pradesh, India. *

**Keywords:** Gallbladder cancer, North Indians, tumor suppressor gene, codon 72, polymorphism

## Abstract

**Background:**

Carcinogen metabolism pathway and tumor suppressor gene polymorphisms have been reported to be associated with increased gallbladder cancer risk. However, the association of genetic variants and gallbladder cancer risk in Indians are not well studied. We examined whether genetic polymorphisms of metabolic enzymes cytochrome P450 1A1 and glutathione S-transferase and tumor suppressor gene p53 (*TP53)* are associated with an increased risk of gallbladder cancer in North Indians.

**Methods:**

This hospital-based case-control study was conducted in 96 gallbladder cancer patients with gallstones (cases) and 93 cholelithiasis patients (controls) at the Sanjay Gandhi Postgraduate Institute of Medical Sciences in Lucknow, India from July 2014 through May 2017. Genomic DNA was extracted from white blood cells of each patient using a simple salting-out procedure. The genotypic frequencies of *CYP1A1* rs4646903, *CYP1A1* rs1048943, and *TP53* rs1042522 polymorphisms were investigated using TaqMan SNP Genotyping Assay and *GSTM1* and *GSTT1* polymorphisms were analyzed using the multiplex PCR assay.

**Results:**

The frequency of CC genotype of TP53 rs1042522 polymorphism was 27.1% (26/96) in cases and 12.9% (12/93) in controls. The CC genotype was associated with an increased risk of gallbladder cancer in North Indians (age- and sex-adjusted odds ratio, 2.81; 95% confidence interval, 1.19–6.61; P = 0.02). No significant differences in genotypic and allelic frequencies of the metabolic pathway gene polymorphisms were found between cases and controls.

**Conclusions:**

Our data provide preliminary evidence that the CC genotype of the *TP53* rs1042522 polymorphism may be associated with an increased risk of gallbladder cancer in North Indians.

## Introduction

Gallbladder cancer is a relatively rare malignancy worldwide (Bray et al., 2018), but the incidence shows marked geographic variation and racial and ethnic variability (Wistuba and Gazdar, 2004). Notably, the incidence of gallbladder cancer in India is high, especially in the northern area (Gupta et al., 2016). Nevertheless, the pathogenic mechanism of gallbladder cancer has not been well identified.

The presence of gallstones in the gallbladder is the most strongly associated risk factor for gallbladder cancer (Sharma et al., 2016). However, only 0.5%–1.5% patients are diagnosed with gallbladder cancer after cholecystectomy among patients with cholelithiasis (Rakić et al., 2014). This evidence suggests that not only gallstones but also other factors, such as environmental chemical pollutants, diet, or other environmental factors, are involved in the development of gallbladder cancer. In addition, gene-environment interactions may also explain the geographic and racial variations in gallbladder cancer incidence.

Genetic variants of genes encoding detoxification enzymes that metabolize environmental chemical pollutants and in tumor suppressor genes have been associated with an increased risk of gallbladder cancer as well as other types of cancer (Srivastava et al., 2011), although some studies have shown conflicting results. Therefore, the higher gallbladder cancer incidence in North India compared with other areas may be potentially explained by these genetic variations. However, the effect of these genetic variants on the development of gallbladder cancer in North Indians has not been well assessed.

Here we conducted a case-control study to assess the contribution of genetic variants to gallbladder cancer risk in North Indians. We investigated the frequencies of polymorphisms in the following genes: cytochrome P450 1A1 (*CYP1A1*), a phase I drug-metabolizing enzyme; glutathione-S-transferase class Mu (*GSTM1*) and glutathione-S-transferase class Tera (*GSTT1*), phase II drug-detoxification enzymes; and tumor suppressor gene p53 (*TP53*). We hypothesized that the frequencies of these genetic variants was higher in gallbladder cancer patients with gallstones compared with cholelithiasis patients.

## Materials and Methods

*Subjects*

We conducted a molecular epidemiological study using a case-control design to investigate genetic factors that affect the risk of gallbladder cancer in North Indians from July 2014 through May 2017. This study included 96 patients diagnosed with gallbladder cancer with gallstones at the Department of Surgical Gastroenterology, Sanjay Gandhi Post Graduate Institute of Medical Sciences (SGPGIMS) in Lucknow, India. The controls were 96 patients diagnosed with cholelithiasis at the same hospital in the same period. Gallbladder cancer and cholelithiasis were confirmed by fine needle aspiration cytology, histopathology, or both. Presence or absence of gallstones was confirmed by ultrasonography. Our cases and controls were from similar ethnic backgrounds. Informed consent was obtained from all patients, and the study protocol was approved by the Ethical Committee of the SGPGIMS (IEC code: 2017-59-CP-96) and Hokuriku University (H28, No. 8).

*Genotyping assay*

Genomic DNA was extracted from white blood cells of each patient using a simple salting-out procedure at the SGPGIMS. The extracted DNA was kept frozen and sent to Niigata University, Japan by maintaining cold chain process, where samples were stored at -20°C until the genetic polymorphism analysis was done.

The genotypic frequencies of *CYP1A1* rs4646903, *CYP1A1* rs1048943, and *TP53* rs1042522 polymorphisms were investigated using TaqMan® SNP Genotyping Assay (Thermo Fisher Scientific Inc., Waltham, MA, USA). *GSTM1* and *GSTT1* polymorphisms were analyzed using the multiplex PCR assay with primers for *GSTM1*, *GSTT1*, and albumin (Arand et al., 1996). For quality control, all genotypes obtained were reconfirmed using the same analysis method, and the results were consistent.

*Statistical evaluation*

All cases and controls were classified either as homozygous carriers of wild-type genotype or carriers of one or two mutant alleles of *CYP1A1* rs4646903, *CYP1A1* rs1048943, and *TP53* rs1042522 genotypes. The genotypes of *GSTM1* and *GSTT1* polymorphisms were classified into two genotypes: non-null genotype (normal) and null genotype.

The frequencies of genotypes and alleles in cases and controls were compared using a contingency table chi-square test or Fisher exact test. The age- and sex-adjusted odds ratios (ORs) and 95% confidence intervals (CIs) of gallbladder cancer were estimated using logistic regression models. A two-tailed p-value less than 0.05 was considered to indicate statistical significance. The genotype distribution of *CYP1A1* rs4646903, *CYP1A1* rs1048943, and *TP53* rs1042522 among controls was compared with that expected based on Hardy-Weinberg equilibrium using the chi-square test. When p-values were 0.05 and over, the samples were estimated to be under the Hardy-Weinberg equilibrium. All statistical analyses were performed using SAS 9.4 software (SAS Institute Inc., Cary, NC, USA) and STATA software (SE 14.2, STATA Corporation, TX, USA).

## Results

In this case-control study, we analyzed data from 96 cases and 93 controls (adequate DNA could not be obtained from three control samples). The mean age of cases (51.6 ± 11.1 years; range, 30–85 years) was not significantly different compared with that of controls (48.6 ± 12.8 years; range, 19–81 years) (P = 0.09). No significant difference in the proportion of female patients was found between cases (58.3%, 56/96) and controls (64.5%, 60/93) (P = 0.38).

Table 1 shows the associations of genotypic and allelic frequencies of *CYP1A1* rs4646903, *CYP1A1* rs1048943, *GSTM1*, and *GSTT1* polymorphisms with gallbladder cancer risk. The genotypic distribution of *CYP1A1* among controls was in Hardy-Weinberg equilibrium: P = 0.31 for rs4646903 and P = 0.11 for rs1048943. No significant differences were found in the genotypic and allelic frequencies of *CYP1A1*, *GSTM1*, and *GSTT1* polymorphisms between cases and controls.

Table 2 shows the association of genotypic and allelic frequencies of the *TP53* polymorphism with gallbladder cancer risk. The frequencies of the three genotypes, GG homozygotes, CG heterozygotes, and CC homozygotes, were 30.2% (29/96), 42.7% (41/96), and 27.1% (26/96) in cases and 37.6% (35/93), 49.5% (46/93), and 12.9% (12/93) in controls, respectively. The *TP53 *genotypic distribution among controls was in Hardy-Weinberg equilibrium (P = 0.60). The CC genotype was associated with an increased risk for the development of gallbladder cancer (age- and sex-adjusted OR, 2.81; 95% CI, 1.19–6.61; P = 0.02). In addition, the frequency of the C allele in cases (48.4%, 93/192) was significantly higher than that in controls (37.6%, 70/186). The presence of the C allele was associated with an increased risk of gallbladder cancer (age- and sex-adjusted OR, 1.57; 95% CI, 1.04–2.37; P = 0.03). However no significant difference in the CG plus CC genotypic frequency was found between cases and controls (age- and sex-adjusted OR, 1.44; 95% CI, 0.78–2.65; P = 0.25).

**Table 1:** Associations of Carcinogen Metabolic Gene Polymorphisms with Gallbladder Cancer Risk

Genotype and allele	Number (%) of		Unadjusted analysis		Multivariate analysis ^a^	
	Cases	Controls	OR (95% CI)	P value	OR (95% CI)	P value
*CYP1A1* rs4646903						
TT	42 (43.8)	37 (40.4)	1		1	
CT	48 (50.0)	47 (50.0)	0.90 (0.50-1.64)	0.73	0.93 (0.51-1.70)	0.81
CC	6 (6.2)	9 (9.6)	0.59 (0.19-1.81)	0.35	0.62 (0.20-1.94)	0.41
CT + CC	54 (56.2)	56 (59.6)	0.85 (0.48-1.52)	0.58	0.88 (0.49-1.58)	0.67
PHWE		0.31				
T	132 (68.8)	121 (65.4)	1		1	
C	60 (31.2)	65 (34.6)	0.82 (0.52-1.31)	0.42	0.85 (0.53-1.36)	0.49
*CYP1A1* rs1048943						
AA	71 (75.5)	63 (70.8)	1		1	
AG	21 (22.3)	26 (29.2)	0.72 (0.37-1.40)	0.33	0.78 (0.93-1.53)	0.46
GG	2 (2.1)	0 (0)	NA	NA	NA	NA
AG + GG	23 (24.5)	26 (29.2)	0.79 (0.41-1.51)	0.47	0.85 (0.44-1.66)	0.64
PHWE		0.11				
A	163 (86.7)	152 (85.4)	1		1	
G	25 (13.3)	26 (14.6)	0.89 (0.48-1.64)	0.71	0.97 (0.52-1.82)	0.93
*GSTM1*						
Non-null	63 (65.6)	62 (66.7)	1		1	
Null	33 (34.4)	31 (33.3)	1.05 (0.57-1.91)	0.88	1.14 (0.61-2.10)	0.69
GSTT1						
Non-null	83 (86.5)	79 (84.9)	1		1	
Null	13 (13.5)	14 (15.1)	0.88 (0.39-2.00)	0.77	0.91 (0.40-2.07)	0.82

^a^Adjusted for sex and age; OR, odds ratio; CI, confidence interval; PHWE, P value for Hardy-Weinberg equilibrium test among controls; NA, not applicable.

**Table 2:** Association between *TP53* rs1042522 Polymorphism and Gallbladder Cancer Risk

Genotype and allele	Number (%) of		Unadjusted analysis		Multivariate analysis ^a^	
	Cases	Controls	OR (95% CI)	P value	OR (95% CI)	P value
GG	29 (30.2)	35 (37.6)	1		1	
CG	41 (42.7)	46 (49.5)	1.08 (0.56-2.06)	0.83	1.09 (0.57-2.10)	0.80
CC	26 (27.1)	12 (12.9)	2.61 (1.13-6.07)	0.03	2.81 (1.19-6.61)	0.02
CG + CC	67 (69.8)	58 (62.4)	1.39 (0.76-2.55)	0.28	1.44 (0.78-2.65)	0.25
PHWE		0.60				
G	99 (51.6)	116 (62.4)	1		1	
C	93 (48.4)	70 (37.6)	1.52 (1.02-2.28)	0.04	1.57 (1.04-2.37)	0.03

^*^Adjusted for sex and age; OR, odds ratio; CI, confidence interval; PHWE, P value for Hardy-Weinberg equilibrium test among controls; NA, not applicable.

## Discussion

Our results demonstrate that the CC genotype of the TP53 polymorphism is associated with higher gallbladder cancer susceptibility in North Indians. However, no significant associations were observed between metabolic pathway gene polymorphisms and gallbladder cancer risk.

Previous studies have examined environmental and genetic risk factors for gallbladder cancer in Indians (Indian Council of Medical Research 2014). Shukla (2001) reported that a risk factor for gallbladder cancer in Indians is the agricultural chemical (organochlorine pesticide) pollution of Ganges River water, similar to the Niigata Prefecture that showed the highest gallbladder cancer incidence in Japan in the 1970s (Yamamoto, 2003). Gene-environment interactions are more strongly related to the incidence of gallbladder disease, including gallbladder cancer, than the effects of environmental factors alone (Weiss et al., 1984). In a study of a Japanese population, genetic variants of genes involved with the metabolism of environmental carcinogens, such as agricultural chemicals, were reported to be associated with an increased risk of gallbladder cancer (Tsuchiya et al., 2007). This evidence suggests an interaction between higher genetic susceptibility to environmental risks and an increased risk of developing gallbladder cancer. If agricultural chemical pollution is associated with an increased risk of gallbladder cancer in Indians, the presence of individuals with the higher genetic susceptibility to metabolic detoxification of agricultural chemicals is suggested.

Some studies have examined the associations between *CYP1A1*, *GSTM1*, and *GSTT1* polymorphisms and gallbladder cancer risk (Pandey et al., 2006; Pandey et al., 2008; Sun et al., 2014), but the findings have been inconsistent. Tsuchiya (2007) reported a positive association between genetic polymorphism of *CYP1A1*, which metabolizes chemical pollutants discharged into the environment, and gallbladder cancer risk in Japanese and Hungarians (Kimura et al., 2008). However, no significant association between *CYP1A1* polymorphism and gallbladder cancer risk were found in Chileans and Bolivians (Tsuchiya et al., 2010; Sakai et al., 2016). The *GSTM1* null genotype was significantly associated with an increased risk of gallbladder cancer in Bolivians (Sakai et al., 2016), though no associations were observed in Japanese, Hungarians, and Chileans (Tsuchiya et al., 2007; Kimura et al., 2008; Tsuchiya et al., 2010). The presence of gallstones in the gallbladder is reported to be a common risk factor for gallbladder cancer worldwide (Goetze, 2015). Gallbladder cancer is a multifactorial disorder, and the development of gallbladder cancer is suggested to be associated with not only gallstones but also other geographically-specific environmental factors. This may be the reason underlying the differences in environmental or genetic risk factors for high gallbladder cancer incidence among countries. To the best of our knowledge, there has been no study on the association between genetic variations of carcinogen metabolic enzymes and the risk of gallbladder cancer in Indians. In this study, we speculated that agricultural chemical pollution in the environment may be a critical risk factor for gallbladder cancer and examined if genetic variants of *CYP1A1*, *GSTM1*, or *GSTT1* were associated with gallbladder cancer risk. We did not observe any significant differences in the frequencies of any genotype between cases and controls. Our data indicate no association between variants in these genes and gallbladder cancer risk, indicating these genetic variants do not play an important role for the development of gallbladder cancer in North Indians.

P53 protein, encoded by *TP53* gene, plays a critical role in cell cycle control, apoptosis, anti-aging, and the maintenance of DNA integrity (Xu and el-Gewely, 2001). The tumor suppressor activity of this protein is explained by its activity to induce apoptosis in response to various stresses. Dumont (2003) reported that the GG genotype (arginine variant) of *TP53 rs1042522* polymorphism induces apoptosis markedly better than the CC genotype (proline variant). The authors showed that the two genotypes of this polymorphism are functionally distinct, and the differences in apoptosis-inducing activity may influence cancer risk or treatment. *TP53 rs1042522* polymorphism has been associated with increased risk of various cancers, including breast cancer, prostate cancer, thyroid cancer, transitional cell carcinoma and chronic myeloid leukemia (Huang et al., 2003; Gemignani et al., 2004; Wu et al., 2004; Granja et al., 2004; Kuroda et al., 2003; Bergamaschi et al., 2004). Our previous studies found that the CG genotype of the *TP53 rs1042522* polymorphism was associated with an increased risk of gallbladder cancer in Japanese men and Hungarians (Tsuchiya et al., 2007; Sakai et al., 2016). To the best of our knowledge, no study has examined the association between the *TP53* polymorphism and gallbladder cancer risk in Indians, so we examined the association in this case-control study. A previous study on the association of the *TP53* polymorphism with the development of cancer in Indians reported that the mean frequencies of the GG, CG, and CC genotypes in controls were 33.1% (range, 14.4%–80.6%), 46.8% (range, 15.1%–65.4%), and 20.1% (range, 4.3%–38.5%), respectively (Mandal et al., 2014). Based on these findings, we regarded the GG genotype or G allele as the major genotype or allele. In the present study, the genotypic frequencies of GG, CG, and CC were similar to those reported previously. Individuals with the CC genotype showed a 2.81-fold significantly higher risk of developing gallbladder cancer than those with the GG genotype. The C allele was also associated with an increased risk of gallbladder cancer, but no significant association was observed between the CG plus CC genotypes and gallbladder cancer risk. These data provide evidence that the CC genotype of the *TP53* polymorphism is associated with an increased risk of gallbladder cancer, and North Indians with the CC genotype have higher susceptibility to gallbladder cancer.

Some limitations should be considered upon interpreting our findings. Our results show a simple statistical association between the CC genotype and an increased risk of gallbladder cancer, but this does not confirm a relation of cause and effect. The reason is that the mutation at codon 72 of *TP53 (rs1042522)* is considered as a gain-of-function gene mutation. However, we speculate that the increased gallbladder cancer risk in the patients with the CC genotype may be explained by the lower apoptosis-inducing activity in the CC genotype than in the GG genotype. Furthermore, the sample sizes for cases and controls were small, so the role of this polymorphism in the pathogenesis of gallbladder cancer may not be accurately represented. We recruited cholelithiasis patients as our controls, but the results may have been clearer if healthy subjects were used as controls. A previous large-scale meta-analysis demonstrated that this* TP53* polymorphism is not associated with any types of cancer risk in Indians (Mandal et al. 2014). However, the study did not examine gallbladder cancer. Thus, this is the first report regarding the role of the *TP53* genetic variant in gallbladder cancer risk in Indians. In contrast, Sahu (2016) reported that both allele frequency and genotype distribution of the *TP53 *polymorphism are associated with an increased risk of nasopharyngeal carcinoma in a meta-analysis. Our findings that the CC genotype is associated with an increased risk of gallbladder cancer should be examined in additional studies with a larger sample size, a meta-analysis, or a systematic review.

In summary, here we found that the CC genotype of *TP53 rs1042522* polymorphism is associated with an increased risk of gallbladder cancer in North Indians in this hospital-based case-control study. The GG genotype of the *TP53* polymorphism was previously shown to have a more potent apoptosis-inducing activity than the CC genotype (Dumont et al., 2003). Carriers with the CC genotype among North Indians appear to show an increased risk of developing gallbladder cancer, which may be due to the lower apoptosis-inducing activity. Our preliminary evidence suggests that the CC genotype of the *TP53 rs1042522* polymorphism is likely to increase the risk of gallbladder cancer in North Indians, but further confirmations with a larger sample size or in studies with healthy subjects as controls are needed.
